# Identification of *TaBADH-A1* allele for improving drought resistance and salt tolerance in wheat (*Triticum aestivum* L.)

**DOI:** 10.3389/fpls.2022.942359

**Published:** 2022-08-01

**Authors:** Ming Yu, Yang Yu, Sihai Guo, Mingfei Zhang, Nan Li, Shuangxing Zhang, Hongwei Zhou, Fan Wei, Tianqi Song, Jie Cheng, Qiru Fan, Caiyin Shi, Wenhan Feng, Yukun Wang, Jishan Xiang, Xiaoke Zhang

**Affiliations:** ^1^College of Agronomy, Northwest A&F University, Xianyang, China; ^2^Academy of Agricultural Sciences, Key Laboratory of Agro-Ecological Protection & Exploitation and Utilization of Animal and Plant Resources in Eastern Inner Mongolia, Chifeng University, Chifeng, China

**Keywords:** wheat, drought, salt, *TaBADH-A1*, allele

## Abstract

Drought and salt stress can strongly affect the growth and development of wheat. Wheat adapts to drought and salt stress through osmotic regulation. Betaine aldehyde dehydrogenase (BADH) is a key enzyme in the synthesis of betaine, an osmotic regulator. We cloned a region of the *TaBADH-A1* promoter and genomic DNA that included the introns and exons, from four Chinese wheat cultivars. Following the analysis of *TaBADH-A1* genomic DNA and promoter sequence polymorphisms of 4 cloned and 15 cultivars from the database, 7 haplotypes of *TaBADH-A1* gene were identified. We divided the 7 haplotypes with a 254 bp insertion or deletion (indel) into two main alleles, *BADH-A1a* and *BADH-A1b*. Meanwhile, a molecular marker was developed based on the 254 bp indel of the third intron of *TaBADH-A1* gene. Expression levels of *BADH-A1b* were found to be significantly higher than those of *BADH-A1a* under drought and salt stress conditions. Betaine accumulation was significantly higher in wheat containing *BADH-A1b* compared to *BADH-A1a* under drought and salt stress. We also identified that the average relative germination and survival rates of wheat with the *BADH-A1b* allele were significantly higher than wheat with the *BADH-A1a* allele. The results reveal that wheat containing *BADH-A1b* has stronger drought and salt tolerance than wheat with *BADH-A1a*. Meanwhile, the geographic distribution and frequency of the *TaBADH-A1* locus alleles indicate that *BADH-A1a* has been preferred in Chinese wheat breeding programs, while *BADH-A1b*, associated with favorable stress tolerance, has been neglected. The results of this study provide evidence for an excellent candidate allele for marker-assisted selection of new wheat cultivars with increased salt tolerance and drought resistance.

## Introduction

It is well known that wheat is one of the most important and widely cultivated cereals, supporting the daily caloric and protein requirements of 60% of the world’s population (Tadesse et al., 2017). Drought has become a serious environmental problem due to global climate change, and a reduction of water by up to 40% causes a reduction in wheat yield of up to 21% (Younis et al., 2020). At the same time, salinization is also an important factor in reduced crop productivity. Salt issues affect about 20% of the total global land area and almost 40% of irrigated lands; this phenomenon shows an increasing trend, and 50% of total cultivated land worldwide is projected to be salinized by 2050 (Urbanavičiūtė et al., 2021). Among all abiotic stresses, stress due to drought and salt is the main challenge for plant growth and development, causing great damage to food production worldwide (Ma et al., 2020). Therefore, improving the drought and salt tolerance is an urgent problem to be solved in wheat breeding. One effective way to improve drought and salt resistance in wheat is to explore and utilize excellent alleles of genes related to drought resistance and salt tolerance.

Both drought and salt stress trigger osmotic regulation in plants, which is the main component of the physiological mechanism determining the drought resistance and salt tolerance of plants (Khoshro et al., 2013; Zhu, 2016). Under drought and salt stress, there is an increased accumulation of substances involved in osmotic regulation, such as betaine, proline, soluble sugar, and inorganic ions, which decreases the osmotic potential and increases the osmotic pressure of cells, resulting in buffering of the redox potential and the maintenance of protein structure, and photosynthetic organs are protected from stress damage (Gadallah, 1999; Mäkelä et al., 2000; Cha-Um and Kirdmanee, 2010; Wang et al., 2018). Betaine, an important osmotic regulatory substance, also has multiple functions in reducing cell osmotic potential and increasing cell osmotic pressure to protect plants from abiotic stress (Zhang et al., 2020). Betaine aldehyde dehydrogenase (BADH) is an important enzyme that catalyzes the final step of betaine production (Fujiwara et al., 2008). The *BADH* gene was first cloned in *Spinacia oleracea* (Weretilnyk and Hanson, 1990) and has, to date been isolated in a large number of plants, such as *Sesuvium portulacastrum*, *Amaranthus hypochondriacus* L., *Arabidopsis thaliana*, and *Triticum aestivum* L. (Legaria et al., 1998; Missihoun et al., 2011; Yang et al., 2015; Sun et al., 2019). Previous studies have shown that *BADH* overexpression in plants can increase their tolerance to various abiotic stresses (Fan et al., 2012; Yang et al., 2015; Sun et al., 2019). For example, overexpression of *SoBADH* (*S. oleracea*) in transgenic sweet potato improved tolerance to salt and oxidative stress and low temperature by increasing betaine accumulation (Fan et al., 2012). Overexpression of *SpBADH* (*S. portulacastrum*) in *Arabidopsis* resulted in higher betaine content and increased tolerance to drought/osmotic stress (Yang et al., 2015). Overexpression of *TaBADH* (*T. aestivum* L.) in *Arabidopsis* was found to enhance betaine accumulation and salt tolerance (Sun et al., 2019). The betaine content and *TaBADH* expression level of different wheat cultivars were found to be significantly different under abiotic stresses such as drought and salt (Jing et al., 1999; Zhao et al., 2001; Li et al., 2005). We speculate that there may be differences in the transcriptional regulatory sequences of the *TaBADH* that lead to the differing betaine contents of the various wheat cultivars.

In the process of evolution, domestication, and breeding, wheat has undergone two doublings, *via* crossing, in reaching its present polyploid form. Since the A genome is the basic genome of wheat evolution, it plays a central role in the evolution of polyploid wheat, contributing to its rich genetic diversity. In this study, we analyzed the *TaBADH-A1* gene sequence polymorphisms in 19 wheat cultivars, evaluated the drought resistance and salt tolerance of *TaBADH-A1* locus alleles, and explored the molecular and physiological mechanisms, and this information was used in the development of a functional molecular marker, 6 AM. We also explored the correlation between *TaBADH-A1* locus alleles and agronomic traits, the distribution of alleles in Chinese wheat zones, and the occurrence frequency of *TaBADH-A1* locus alleles in wheat breeding years. As a result of our study, we present an excellent candidate allele for the molecular breeding of wheat with improved drought and salt tolerance.

## Materials and methods

### Plant materials

Four Chinese wheat cultivars (Taishan 1, Zhongmai 9, Bima 4, and Lantian 23) with different levels of adaptability were selected for cloning *TaBADH-A1*. Taishan 1 has the excellent characteristic of strong resistance to stress; Zhongmai 9 withstood the rare occurrence of freezing damage in 1993–1994 and exhibits strong resistance to abiotic stresses; Bima 4 is one of the backbone parents of wheat in China with a large cultivation area, and it is planted in regions with sufficient water and fertilizer; and Lantian 23 is a high-yielding cultivar adapted to abundant water and fertile conditions. A set of nulli-tetrasomic lines of Chinese Spring (N6AT6B, N6BT6D, and N6DT6A) was used as PCR template, agarose gel electrophoresis results as a reference for the chromosome localization of primers. A total of 121 wheat cultivars (lines; population 1) in different ecological regions covering 10 wheat-producing areas in China were selected for the identification of drought resistance at the germination stage to evaluate the drought resistance of different alleles. Using Chinese Spring and Yanfu 188 as parents, a population of F_6_ recombinant inbred lines (population 2) was constructed to verify the drought and salt resistance of *TaBADH-A1* locus different alleles. Similarly, another population of F_6_ recombinant inbred lines (population 3) was created using Chinese Spring and Ning 9940; two F_6_ populations were obtained using the single-seed descent approach. The relationship between *TaBADH-A1* locus alleles and agronomic traits was validated in both populations. A wheat mini-core collection (population 4, 262 cultivars (lines; landraces), provided by Chenyang Hao at the Chinese Academy of Agricultural Sciences), which is characterized by wide geographic distribution, was used for geographic distribution analysis of the *TaBADH-A1* locus alleles. In addition, 121 cultivars (lines; population 1) also were selected to analyze the geographic distribution and frequency of *TaBADH-A1* locus alleles in breeding years.

### DNA extraction, *TaBADH-A1* gene cloning, and sequence analysis

Genomic DNA of the 4 wheat cultivars (Taishan1, Bima 4, Zhongmai 9, and Lantian 23) and 4 populations was extracted using the CTAB method (Stein et al., 2010). *TaBADH-A1*-specific primers were designed (Supplementary Table S1) according to the assembled *TaBADH-A1* sequences (*TraesCS6A02G371100*) using Oligo 7.0 software. Subsequently, a region of the promoter and genomic DNA sequence that includes the introns and exons of *TaBADH-A1* was cloned from each of the 4 cultivars. The PCR system details are as follows: 50 μl reactions contained 2 μl each of forward and reverse primers (10 μM) and 2 μl DNA (300 ng·μL^−1^), 25 μl of 2 × Phanta Max Buffer, 1 μl dNTP Mix, 1 μl Phanta Max Super-Fidelity DNA Polymerase, and 17 μl ddH_2_O. The PCR program comprised a cycle of 95°C for 3 min followed by 34 cycles of 95°C for 15 s, 62°C for 15 s, and 72°C for 8 min, followed by 95°C for 15 s, 62°C for 15 s, and 72°C for 8 min, and finally 72°C for 5 min (Phanta Max Super-Fidelity DNA Polymerase, Vazyme).

Sequences for the *TaBADH-A1* promoter and genomic region including introns and exon of 15 wheat cultivars (ArinaLrFor, Cadenza, Claire, Jagger, Julius, Lancer, Paragon, SY Mattis, Robigus, Weebill, Stanley, Landmark, Norin 61, Mace, and Chinese Spring) were downloaded from the Ensembl Plants database^1^. The *TaBADH-A1* gene sequence polymorphisms were analyzed in Taishan 1, Bima 4, Zhongmai 9, Lantian 23, and the 15 wheat cultivars. To analyze the *TaBADH-A1* sequences of genomic (include intron and exon) and promoter regions, the Giri^2^ and PlantCare^3^ databases were used for the prediction of transposons and *cis*-acting elements. DNAMAN 9.0 software was used for sequence alignment.

### Development of a molecular marker

After analyzing the *TaBADH-A1* gene sequence polymorphism in Taishan 1, Bima 4, Zhongmai 9, Lantian 23, and 15 wheat cultivars based on sequences from the database, the molecular marker 6 AM was developed based on the insertion/deletion (indel) of 254 bp in the third intron region. Primers for the molecular marker 6 AM were designed as 5′-CGCAAGTCAATGGGCTGTGTAT-3′ and 5′-CAAGACAGGAGGGGAATGGGAAAT-3′. The PCR system components were as follows: 10 μl of 2× Rapid Taq Master Mix, 7 μl ddH_2_O, 1 μl 6 AM-F (10 μM), 1 μl 6 AM-R (10 μM), and1 μL DNA (300 ng·μL^−1^; Rapid Taq Master Mix, Vazyme). The PCR program for 6 AM comprised a cycle at 95°C for 3 min, 95°C for 15 s, 62°C for 15 s, and 72°C for 15 s, followed by 34 additional cycles of 95°C for 15 s, 62°C for 15 s, and 72°C for 15 s, and finally 72°C for 5 min. The PCR products were separated on 2% agarose gel. The primer pairs were located by PCR amplification using the nulli-tetrasomic lines of Chinese Spring as a template. The validity of the 6 AM primers was verified using PCR-based amplified fragment length polymorphism. Genotyping was performed in population 1, 2, 3, and 4 using the 6 AM molecular marker to characterize the function of alleles.

### RNA extraction and qRT-PCR analysis

The 5 randomly selected recombination inbred lines (population 2), Yanfu 188, and Bima 4 with the *BADH-A1a* allele and the 5 randomly selected lines of population 2, Taishan 1, and Chinese Spring with the *BADH-A1b* allele were planted in half-strength Hoagland nutrient solution in a light incubator at 22°C under controlled conditions (60% relative humidity, 16/8 h light/dark cycle). At 4-leaf stage seedlings, Yanfu 188, Bima 4, Taishan 1, and Chinese Spring leaf samples were collected at untreated 0, 1, 6, 12, and 24 h. Subsequently, all wheat at 4-leaf stage seedlings were treated with half-strength Hoagland nutrient solution containing 200 mM NaCl or 20% PEG 6000. Leaf samples were collected at treated 0, 1, 6, 12, and 24 h. All samples were immediately stored at −80°C for further RNA extraction.

Total RNA was extracted using TRIzol (Qingke, Beijing), and cDNA was then synthesized using EasyScript One-Step gDNA Removal and cDNA Synthesis SuperMix (TransGen Biotech, Beijing). Quantitative real-time PCR (qRT-PCR) was performed using Perfect Start™ Green qPCR Supermix (TransGen Biotech, Beijing). *TaActin* was used as an internal reference gene for wheat. All qRT-PCR analyses were conducted in this study based on 3 technical replicates and 3 biological replicates. Relative gene expression levels were calculated using the 2^−ΔΔCt^ method (Ma et al., 2020).

### Betaine content determination

The materials used for expression analysis, Ning 9940 with *BADH-A1a* allele and Longfumai 18 with *BADH-A1b* allele, were grown and treated in the same way as above (RNA extraction and qRT-PCR analysis section). Leaf samples were collected from Longfumai 18, Taishan 1, Chinese Spring, Bima 4, Yanfu 188, and Ning 9940 to determine betaine content after plants had undergone stress treatments for 0, 1, 6, 12, 24, and 48 h with half-strength Hoagland nutrient solution containing 200 mM NaCl or 20% PEG 6000. Similarly, population 2 leaf samples were collected after stress treatment for 48 h with half-strength Hoagland nutrient solution containing 200 mM NaCl or 20% PEG 6000. All the samples were dried, each 0.2 g dry weight sample was thoroughly mixed with 1 mL of 80% methanol at 60°C for 30 min and centrifuged at 10,000 rpm for 15 min, and the supernatant from the sample that had absorbed methanol was completely volatilized to a constant volume of 1 ml as the sample solution. Betaine solutions at 0.1, 0.2, 0.3, 0.4, 0.5, 0.6, 0.7, 0.8, 0.9, and 1.0 mg·L^−1^ were prepared as standard solutions, with ddH_2_O as the blank or negative control. Then, 0.1 mL each of sample solution, standard solution, and ddH_2_O was thoroughly and separately mixed with 1 ml of saturated Reinecke salt solution (pH = 1) and incubated at 4°C for 2 h, centrifuged at 8000 rpm for 15 min, then the supernatant was removed and mixed with ether, followed by centrifuging at 8000 rpm for 15 min, and the supernatant from this step was then mixed with 70% acetone. The absorbance of this mixture was then measured at 525 nm. A standard curve was drawn using the absorbance values of the standard solutions. The experimental method and the formula for calculation of betaine content were used as indicated in the instructions of the Solarbio betaine content detection kit (BC3130). Three biological and technical replicates were analyzed for each condition.

### Identification of drought resistance and salt tolerance in wheat

The drought resistance of 121 cultivars (lines; population 1) at the germination stage was characterized. A total of 100 seeds were placed into a 12 × 12 × 5 cm^3^ germinating box, and single-layer filter paper was used as the sprout bed. Then, 12 mL of 20% PEG 6000 was added to the treatment group and 12 mL of ddH_2_O was added to the control group. A further 100 seeds were used as replicates, and the experiments for the treatment and control groups were repeated 4 times each. The standard for wheat seed germination was defined as the point at which the length of the wheat radicle was equal to the length of the seed and the length of the germ reached half the length of the seed. The number of germinated seeds was investigated, and the relative germination rate of seeds was calculated after 168 h of culture at 20°C in the dark as a ratio of the germination rate of the treatment group divided by that of the control group.

Five lines with *BADH-A1a* and five lines with *BADH-A1b* from the recombination inbred lines (population 2) were planted in pots with nutritive soil (matrix of soil: vermiculite = 3:1) in a light incubator. The flowerpot dimensions were 10 × 10 cm^2^ at the top, 7.5 × 7.5 cm^2^ at the bottom, and 7.5 cm high, and each pot was filled with 165 g of nutritious soil and planted with 4 wheat seedlings. Drought and salinity stress treatments were imposed at the 4-leaf stage. Under drought treatment, wheat plants were deprived of water for 15 days and then re-watered for 5 days. Under salt stress treatment, wheat plants were irrigated with H_2_O solution containing 700 mM NaCl for 25 days. The ultimate number of surviving plants and survival rates were determined. Each experiment (with each line including 36 wheat plants) was repeated 3 times.

### Field management and investigation of agronomic traits

In 2020, a total of 194 lines from population 2 and 182 lines from population 3 were planted in Yangling (34°16’ N, 108°4′ E; Shaanxi Province, China). Each line was planted in one 0.5 m row, with about 20–30 plants in each row, spaced 25 cm apart in each row. Compound (20% nitrogen, 18% phosphorus, 5% potassium; 300 kg/ha) and nitrogen (46% nitrogen; 300 kg/ha) fertilizers were applied to the wheat before planting. The field was irrigated in December 2020. Data on agronomic traits, including grain number per spike (GN), effective spikelet number per spike (ESN), thousand kernel weight (TKW), flag leaf area per plant (FLA), spike length (SL), length of last internode (LLI), and plant height (PH), were collected from populations 2 and 3. Three plants from each strain were randomly selected to measure GN, ESN, FLA, SL, LLI, and PH. The average values of the measurements were taken as the value for each agronomic trait. Spikelets containing grains were defined as effective spikelets. FLA was calculated as 0.7 × leaf length × leaf width. SL, LLI, and PH were measured from the bottom to the top of the spike, from the last internode to the bottom of the spike, and from ground level to the tip of the spike, respectively.

### Association analysis

Genotyping of populations (1, 2, 3, and 4) was performed using the molecular marker 6 AM. Recombinant inbred lines (populations 2 and 3) were used to analyze the correlations between alleles and agronomic traits. The wheat mini-core collection (population 4) and 121 cultivars (lines) (population 1) were used to analyze the geographic distribution of different alleles, and the frequency of different alleles in wheat releasing years was analyzed using population 1.

### Statistical analysis

The statistically significant differences in *TaBADH-A1* expression level, betaine content, and phenotype between *BADH-A1a* and *BADH-A1b* were evaluated in SPSS 20 software.

## Results

### Sequence polymorphism and molecular marker development of *TaBADH-A1* in wheat

**Figure 1:**
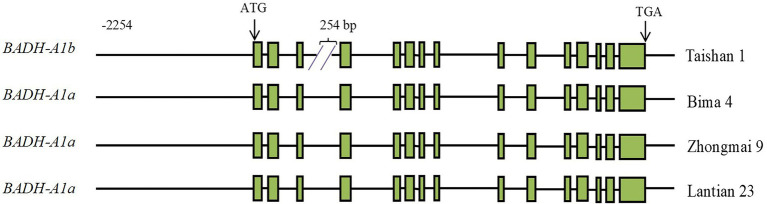
Schematic diagram of *TaBADH-A1* gene structure. ATG start codon is designated as position 1 bp. Green rectangles are exons; black line before ATG is promoter region; black lines after ATG and before TGA are intron regions; black line after TGA is 3′ untranslated region.

A single pair of *TaBADH-A1*-specific primers were designed (Supplementary Table S1) to clone the region of promoter and genomic DNA containing introns and exons of *TaBADH-A1* from the wheat cultivars Taishan 1, Bima 4, Zhongmai 9, and Lantian 23. Based on comparisons with the coding sequence (*TraesCS6A02G371100*), the sequence of *TaBADH-A1* was found to consist of 15 exons and 14 introns (Figure 1). The length of promoter and genomic DNA that includes introns and exons of *TaBADH-A1* in Taishan 1, Bima 4, Zhongmai 9, and Lantian 23 was 7,675, 7,919, 7,919, and 7,919 bp, respectively. The *TaBADH-A1* gene sequence polymorphisms were analyzed in the 4 cloned cultivars and 15 cultivars from the database; we found 42 single nucleotide polymorphisms (SNPs) and four 1 bp and two 2 bp indels in the promoter region of *TaBADH-A1* (Supplementary Table S2), and 28 SNPs, a 254 bp, two 1 bp, and a 2 bp indels in the genomic DNA. The *TaBADH-A1* gene variations were grouped into 7 haplotypes (Supplementary Table S2). Haplotypes 3–7 had 2 additional ABREs, 1 additional I-box, 1 additional O_2_-site, 1 additional G-box, 1 additional STRE, 1 additional TCA-element, 1 additional W-box, 1 additional AT-TATA-box, and 1 less TGA element than haplotypes 1 and 2 in their promoter region. In the third intron region, the 254 bp fragment of insert, which was a DNA miniature inverted-repeat transposable element (MITE), contained 1 Box-4, 1 MRX, 2 W-box, 2 CAAT-box, and 11 TATA-box elements. The SNPs in exons do not lead to amino acid mutations. As DNA MITE can have a greater impact on gene function, we divided the 7 haplotypes with 254 bp indel fragments into 2 main alleles, *BADH-A1a* and *BADH-A1b* (Figure 1; Supplementary Table S2).

Subsequently, specific primers were designed according to sequences at both ends of the 254 bp indel of the *TaBADH-A1* third intron (Supplementary Table S1). DNA of Chinese Spring, N6AT6B, N6BT6D, and N6DT6A and ddH_2_O was used as templates for PCR using molecular marker primers. The PCR products using N6AT6B DNA and ddH_2_O as a template did not contain the target gene, while the other PCR products all contained the target gene (Supplementary Figure S1). The 6 AM marker primers amplify a region located in wheat chromosome 6A. Genotyping of all populations using a molecular marker showed the presence of amplified fragment length polymorphisms in PCR products, indicating the effectiveness of 6 AM as a molecular marker (Supplementary Figure S2). The amplified fragments of 6 AM were 828 and 574 bp, which correspond to *BADH-A1a* and *BADH-A1b*, respectively (Supplementary Figure S2). The functional molecular marker 6 AM was developed based on the 254 bp indel in *TaBADH-A1* (Supplementary Table S1).

### Expression analysis of *TaBADH-A1* locus two alleles in wheat under drought and salt stress

**Figure 2:**
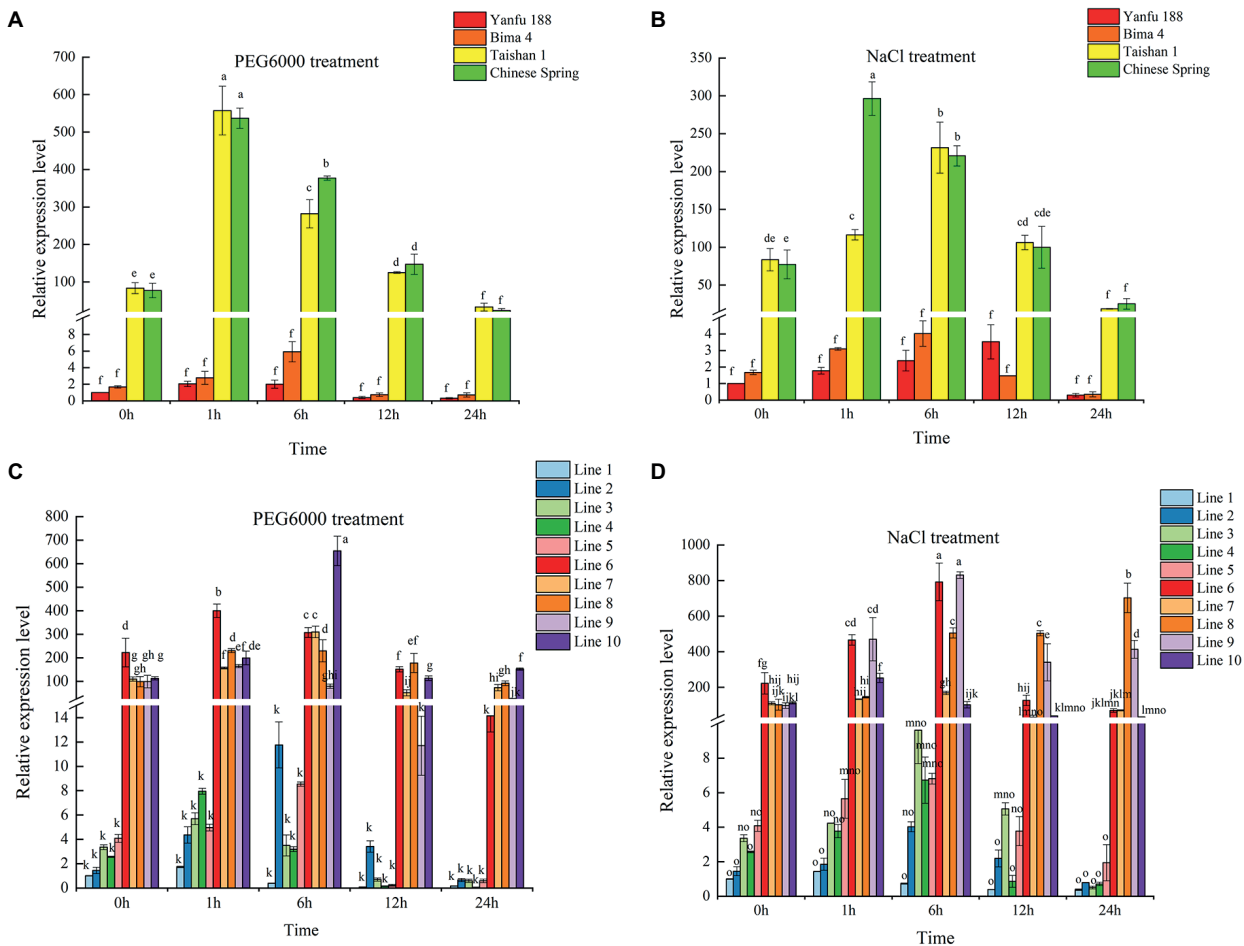
Expression patterns of *TaBADH-A1* locus alleles in salt (200 mM NaCl) and drought (20% PEG 6000). Wheat was subjected to drought and salt stress at 4-leaf stage; leaves were used as samples for RNA extraction. **(A,B)** *TaBADH-A1* expression of Yanfu 188 under control treatment was used as standard. Yanfu 188 and Bima 4 containing *BADH-A1a*; Taishan 1 and Chinese Spring containing *BADH-A1b*. **(C,D)** *TaBADH-A1* expression of line 1 under control treatment was used as standard; lines 1–5 containing *BADH-A1a*; lines 6–10 containing *BADH-A1b*. The 2^−ΔΔCT^ method was used to calculate relative gene expression. Statistically significant differences are indicated with different letters (LSD, *p* < 0.05).

DNA MITE insertion in the third intron might affect gene expression (Dumesic et al., 2013; Kim Guisbert and Sontheimer, 2013). At the 4-leaf stage, Yanfu 188 and Bima 4 with *BADH-A1a* and Taishan 1 and Chinese Spring with *BADH-A1b* were selected to investigate the expression of the two alleles. Under normal conditions, the expression pattern of *TaBADH-A1* gene in the 4 cultivars was consistent, which showed no change in expression level at 0, 1, 6, 12, and 24 h (Supplementary Figure S3). Subsequently, the expression level of the *TaBADH-A1* gene at 0 h of treated can be representative of the expression level of 1, 6, 12, and 24 h of untreated. Under stress treatment for 0 h, the expression level of *BADH-A1b* gene was higher than that of *BADH-A1a*. The expression pattern of *TaBADH-A1* gene in the 4 cultivars was consistent, first increasing and then decreasing with the extension of the drought and salt stress time (Figures 2A,B). Notably, we found that expression levels of *BADH-A1b* were all significantly higher than those of *BADH-A1a* before and after stress (Figures 2A,B). Considering the differences in the genetic background among wheat cultivars, the population of F_6_ recombinant inbred lines (population 2) were constructed using Chinese Spring and Yanfu 188 as parents to further verify the above result. Lines 1–5 of population 2 with *BADH-A1a* and lines 6–10 of population 2 with *BADH-A1b* were selected for further study. Under drought and salt stress, the expression of *TaBADH-A1* gene was found to first increase and then decrease (Figures 2C,D). Meanwhile, expression levels of *BADH-A1b* were significantly higher than those of *BADH-A1a* before and after stress treatment (Figures 2C,D). Therefore, the deletion of DNA MITE can enhance *TaBADH-A1* gene expression.

### Analysis of betaine content in wheat containing different alleles under drought and salt stress

**Figure 3:**
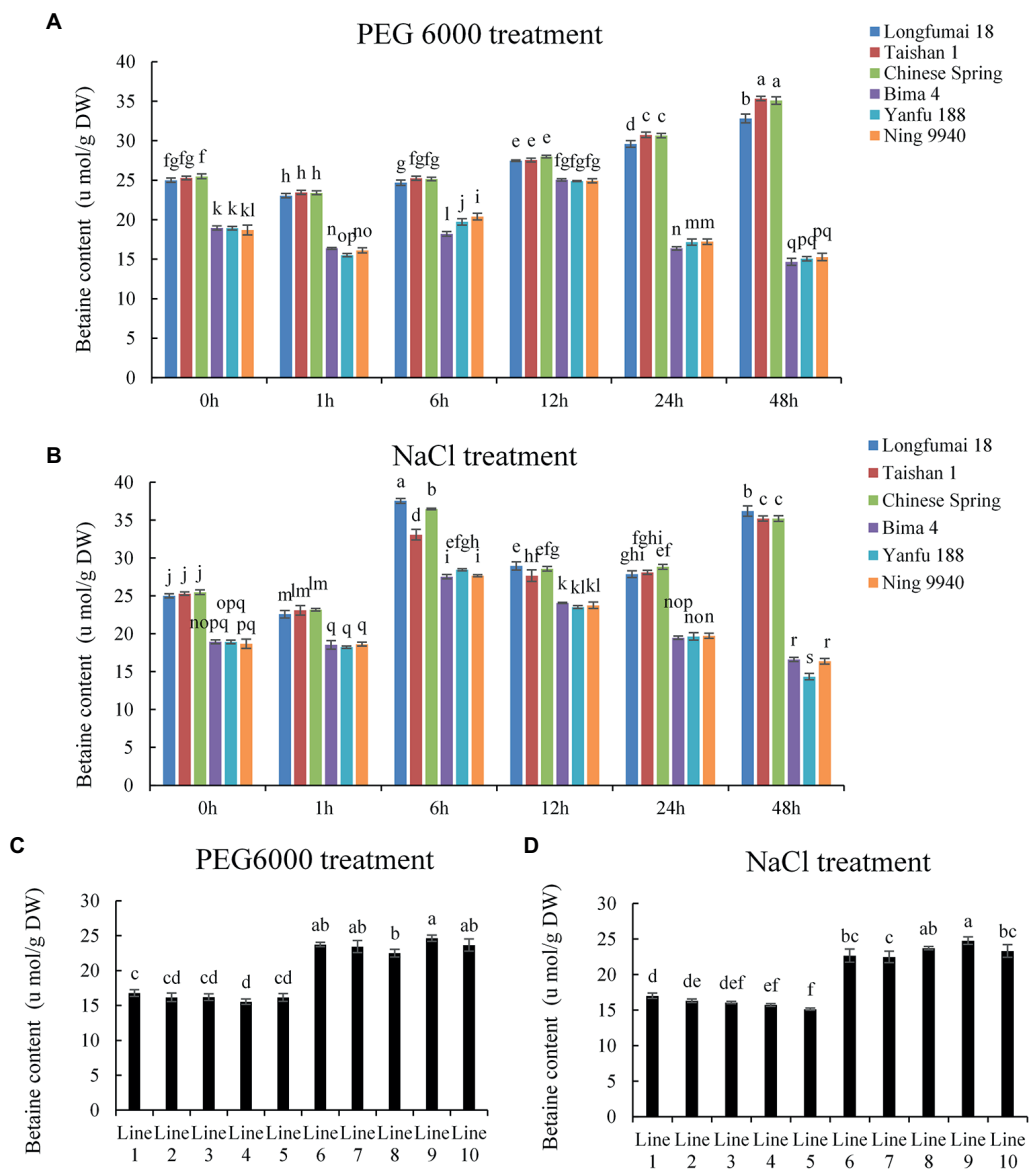
Betaine content of wheat containing different alleles under drought (20% PEG 6000) and salt stress (200 mM NaCl) at 4-leaf stage. DW, dry weight. **(A,B)** Bima 4, Yanfu 188, and Ning 9940 contain *BADH-A1a*; Longfumai 18, Taishan 1, and Chinese Spring containing *BADH-A1b*. **(C,D)** Betaine content of wheat under stress for 48 h; lines 1–5 containing *BADH-A1a*; lines 6–10 containing *BADH-A1b*. Statistically significant differences are indicated with different letters (LSD, *p* < 0.05).

Through genotyping of the 6 AM molecular marker, wheat cultivars with *BADH-A1b* (Longfumai 18, Taishan 1, Chinese Spring) and wheat cultivars with *BADH-A1a* (Bima 4, Yanfu 188, Ning 9940) were selected to identify betaine content under drought and salt stress at the 4-leaf stage. Under drought stress, we found that the betaine content in leaves of wheat cultivars containing *BADH-A1b* decreased slightly at 1 h and then increased gradually, while the content in cultivars containing *BADH-A1a* decreased slightly at 1 h, then increased and peaked at 12 h, and then began to decline (Figure 3A). Under salt stress, betaine content in the leaves of wheat cultivars containing *BADH-A1a* and *BADH-A1b* decreased slightly at 1 h, increased and peaked at 6 h, and then began to decline. However, the betaine content of wheat cultivars containing *BADH-A1b* increased again at 48 h (Figure 3B). It is noteworthy that the betaine content in leaves of wheat cultivars containing *BADH-A1b* was always higher compared to cultivars containing *BADH-A1a* regardless of drought or salt stress, and the largest difference was at 48 h of stress. This might be due to the fact that the expression level of *BADH-A1b* gene was always higher than that of *BADH-A1a*. Furthermore, we used recombinant inbred lines (population 2) to identify the betaine content of lines containing different alleles after 48 h of drought and salt stress. Under drought stress 48 h, the betaine content of lines 6–10, containing *BADH-A1b*, was 24, 23, 23, 25, and 24 μmol·g^−1^ (dry weight, DW), respectively, and the betaine content of lines 1–5, containing *BADH-A1a*, was 17, 16, 16, 16, and 16 μmol·g^−1^ (DW), respectively (Figure 3C). Under salt stress 48 h, the betaine content of lines 6–10 was 23, 22, 24, 25, and 23 μmol·g^−1^ (DW), respectively, and the content of lines 1–5 was 17, 16, 16, 16, and 15 μmol·g^−1^ (DW), respectively (Figure 3D). Therefore, wheat containing *BADH-A1b* had significantly higher betaine content than wheat with *BADH-A1a* under drought and salt stress.

### Analysis of drought and salt resistance of wheat with different alleles

We identified the relative germination rate of 121 cultivars (lines; population 1). The average relative germination rate of wheat containing *BADH-A1a* was 51.2%, while that of *BADH-A1b* was 67.3% (Table 1). The wheat cultivars (lines) containing *BADH-A1b* had a significantly higher average relative germination rate than those with *BADH-A1a*, which shows that *BADH-A1b* can increase the drought resistance of wheat during the germination period.

**Table 1:** Relative germination rate of *TaBADH-A1* locus alleles in 121 cultivars (lines).

Alleles	Cultivar (lines) number	Relative germination rate (%)	
		Average	Range
*BADH-A1a*	109	51.2b	91.8–15.5
*BADH-A1b*	12	67.3a	92.8–45.3
Total	121	52.8	15.5–92.8

Significant differences are indicated with different letters in average relative germination rate (LSD, *p* < 0.05).

**Figure 4:**
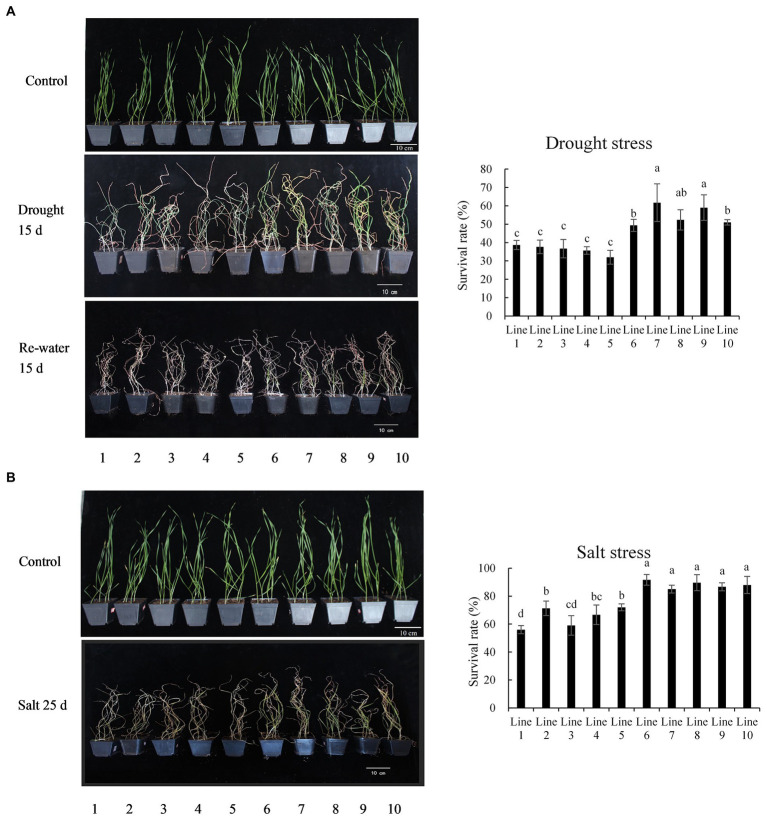
Phenotype analysis of recombinant inbred lines of *TaBADH-A1* locus alleles under drought and salt stress. Lines 1–5 containing *BADH-A1a*; lines 6–10 containing *BADH-A1b*. **(A)** Survival rates calculated after withholding water for 15 days and water recovery for 5 days. **(B)** Survival rates were calculated under salt solution (700 mM NaCl) irrigation for 25 days. Statistically significant differences are indicated with different letters (LSD, *p* < 0.05).

The recombinant inbred lines (population 2) containing different alleles were treated with drought and salt in the 4-leaf stage. Under drought stress, the effects on lines 6–10, containing *BADH-A1b*, were relatively more mild (grew well with green leaves) than lines 1–5, containing *BADH-A1a* (Figure 4A). After 5 days of recovery water treatment, the survival rates of lines 6–10 were 49.3, 61.7, 52.3, 59.0, and 51.0%, respectively, while the survival rates of lines 1–5 were 38.7, 37.7, 36.7, 35.7, and 32.0%, respectively (Figure 4A). The results indicate that *BADH-A1b* could significantly increase the drought resistance of wheat at the 4-leaf stage. After 25 days of salt stress, leaves of lines 1–5, containing *BADH-A1a*, were more withered than those of lines 6–10, with *BADH-A1b*; the survival rates of lines 1–5 were 56.0, 71.3, 59.0, 66.7, and 72.0%, respectively, while the survival rates of lines 6–10 were 91.7, 85.0, 89.7, 86.7, and 88.0%, respectively (Figure 4B). The results show that *BADH-A1b* can significantly increase the salt tolerance of wheat at the 4-leaf stage.

### Association of *TaBADH-A1* locus alleles with agronomic traits

The recombinant inbred line populations (2 and 3) were genotyped according to PCR amplified fragment length polymorphisms using the 6 AM molecular marker. Subsequently, relationships between *TaBADH-A1* locus alleles and agronomic traits were analyzed, including grain number per spike (GN), effective spikelet number per spike (ESN), thousand kernel weight (TKW), flag leaf area per plant (FLA), spike length (SL), length of the last internode (LLI), and plant height (PH). We found no significant differences in GN, ESN, TKW, FLA, SL, LLI, and PH between plants carrying either *BADH-A1a* or *BADH-A1b* (Supplementary Table S3).

### Geographic distribution of *BADH-A1a* and *BADH-A1b* in Chinese zones

**Figure 5:**
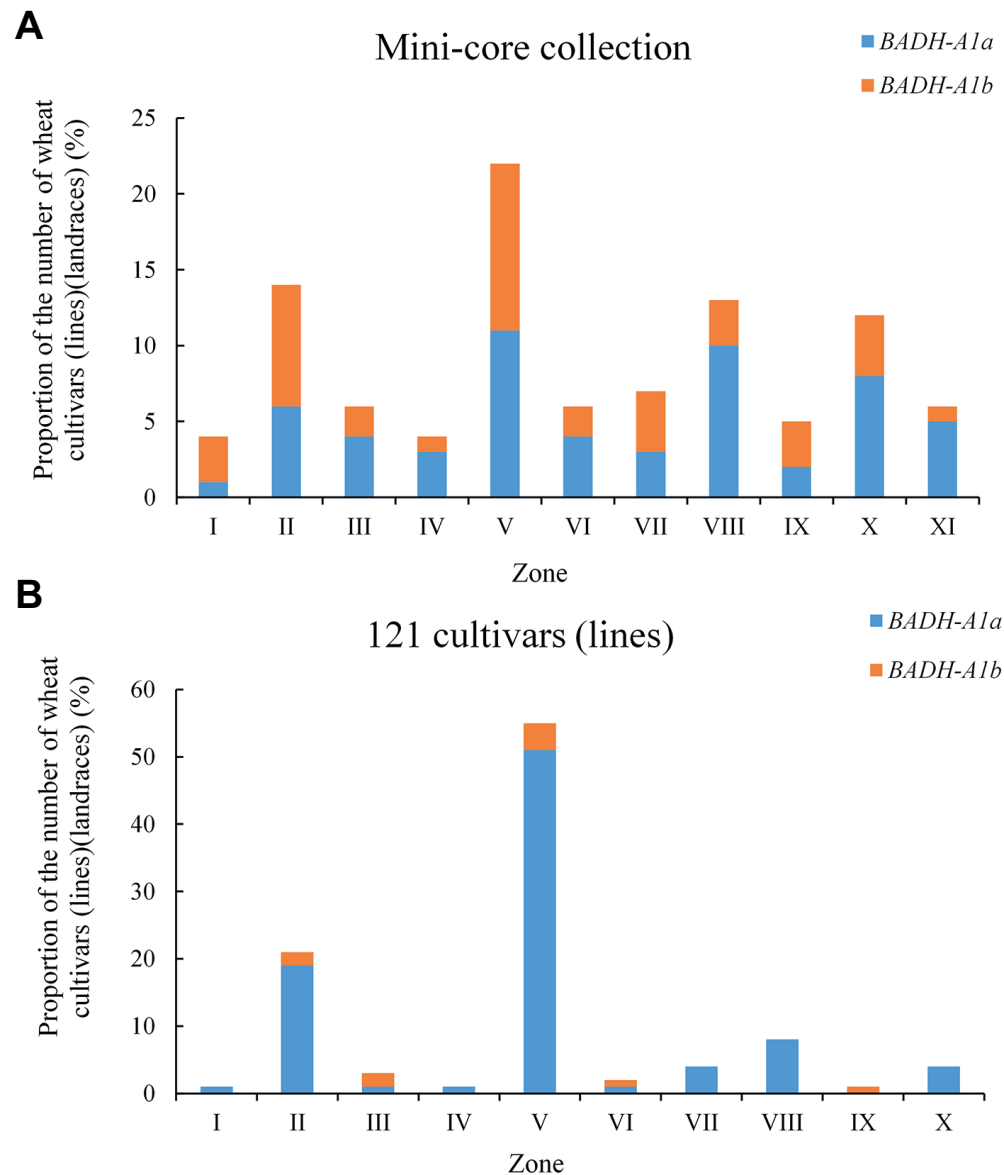
Geographic distribution of *BADH-A1a* and *BADH-A1b* in Chinese wheat production zones. **(A)** In mini-core collection population; **(B)** In 121 cultivars (lines) population. I, Northern Spring Wheat Zone; II, Northern Winter Wheat Zone; III, Northeastern Spring Wheat Zone; IV, Southern Autumn-Sown Spring Wheat Zone; V, Yellow and Huai River Valley Facultative Wheat Zone; VI, Qinghai-Tibetan Plateau Spring–Winter Wheat Zone; VII, Northwestern Spring Wheat Zone; VIII, Southwestern Autumn-Sown Spring Wheat Zone; IX, Xinjiang Winter–Spring Wheat Zone; X, Middle and Lower Yangtze Valley Autumn-Sown Spring Wheat Zone; XI, Overseas introduction.

The wheat production area in China is divided into 10 major agro-ecological production zones based on cropping season, variety type, and ecological conditions (Li et al., 2016). The mini-core collection and 121 cultivars (lines; populations 1 and 4) covering all 10 zones were used to evaluate the geographic distribution of *TaBADH-A1* locus alleles. Overall, *BADH-A1a* and *BADH-A1b* were distributed in all zones (Figure 5A). *BADH-A1a* was present in 59% of the mini-core collection (population 4), while *BADH-A1b* was present in 41% (Figure 5A). However, not all wheat zones had more plants with *BADH-A1a* than *BADH-A1b* in population 4, only the Northeastern Spring Wheat Zone, Southern Autumn-Sown Spring Wheat Zone, Qinghai-Tibetan Plateau Spring–Winter Wheat Zone, Southwestern Autumn-Sown Spring Wheat Zone, Middle and Lower Yangtze Valley Autumn-Sown Spring Wheat Zone, and more overseas introduction plants had *BADH-A1a* than *BADH-A1b*. In population 1, *BADH-A1a* accounted for the vast majority at 90%. The coverage of *BADH-A1b* was only slightly higher than *BADH-A1a* in the Northern Winter Wheat Zone, Northeastern Spring Wheat Zone, and Xinjiang Winter–Spring Wheat Zone (Figure 5B). The results show a weak selection of the favorable allele, *BADH-A1b*, in all zones.

### Frequency of *TaBADH-A1* locus alleles in wheat breeding history

To assess *TaBADH-A1* locus allele frequency in different breeding years, the 121 cultivars (lines) were divided into 6 subgroups according to whether their release dates were in the 1950s, 1960s, 1970s, 1980s, or 1990s, or post-2000. *BADH-A1a* was present in 90% of the 121 cultivars (lines) and all 6 subgroups (Table 2). *BADH-A1b*, which first appeared in the 1980s and reached its peak in the post-2000s, accounted for only 10%, which indicates the allele *BADH-A1b* was selected by breeders to resist progressively deteriorating climatic conditions.

**Table 2:** *TaBADH-A1* locus allele frequency in population 1 wheat breeding years (%).

Years	*BADH-A1a*	*BADH-A1b*	Total
1950s	2	0	2
1960s	3	0	3
1970s	3	0	3
1980s	9	3	12
1990s	20	2	21
Post-2000s	52	5	57
Total	90	10	100

## Discussion

### Analysis of the mechanism of allele *BADH-A1b* in improving drought and salt tolerance in wheat

Osmotic adjustment is the main component of the physiological machinery of drought and salt tolerance in wheat (Khoshro et al., 2013). Betaine, a small, nontoxic organic metabolite, functions as an osmotic regulator under drought and salt stress (Yang et al., 2015). Betaine aldehyde dehydrogenase (BADH) is an important enzyme that catalyzes the last step of betaine synthesis (Fujiwara et al., 2008). In this study, the *TaBADH-A1* gene, which encodes BADH, was cloned in Taishan 1, Bima 4, Zhongmai 9, and Lantian 23. Based on sequence polymorphism analysis, we found 7 haplotypes of the *TaBADH-A1* gene in the 4 cloned wheat cultivars and 15 wheat cultivars from the database. We divided the seven haplotypes with 254 bp indel fragments into two main alleles, *BADH-A1a* and *BADH-A1b*, and the molecular marker 6 AM was developed based on these fragments.

First, the 254 bp fragment inserted into the third intron of *BADH-A1a* contains a TATA-box and W-box. A TATA-box is the core promoter element that initiates gene transcription and participates in the formation of the transcription initiation complex (Bernard et al., 2010). The WRKY transcription factors can bind to W-box, while most of the WRKY transcription factors of known function are negative regulators, with only a few of those characterized as having a positive regulatory role (Jiang et al., 2017). The WRKY transcription factors may recruit other proteins to bind to the W-box of the fragment inserted into the third intron of *BADH-A1a* to jointly inhibit *BADH-A1a* expression. Introns were previously considered useless gene sequences, but they have since been shown to regulate gene expression. For example, the adaptor protein MINI ZINC FINGER 2 binds to the *WUSCHEL* (*WUS*) intron and recruits KNUCKLES, TOPLESS, and HISTONE DEACETYLASE 19 to form a transcriptional repressor complex that represses *WUS* expression in *A. thaliana* (Bollier et al., 2018).

Second, pre-mRNAs containing transposons are subject to inefficient splicing (Dumesic et al., 2013). The introns that contain transposons exhibited a greater dwell time on spliceosomes, and pre-mRNAs in the stalled spliceosomes became substrates for the synthesis of siRNAs used to dampen expression of the corresponding gene (Kim Guisbert and Sontheimer, 2013). The 254 bp fragment inserted into the third intron of *BADH-A1a* contain DNA miniature inverted-repeat transposable elements, which might reduce splicing efficiency and thus suppress *BADH-A1a* expression. The above two processes may be responsible for the higher expression level of *BADH-A1b* than *BADH-A1a* before and after stress.

*BADH-A1b* was always found to be expressed at a high level, which results in increased betaine content. After 48 h of stress, leaves of wheat containing *BADH-A1a* had decreased betaine content, while leaves of wheat containing *BADH-A1b* maintained a higher betaine content. We also identified that the average relative germination and survival rates of wheat with *BADH-A1b* allele under drought and salt stress were significantly higher compared to wheat with *BADH-A1a* allele. *BADH-A1b* which can increase the content of betaine may decrease the osmotic potential and increase the osmotic pressure of cells for over a longer period of stress time more markedly than *BADH-A1a*, further enhancing the drought resistance and salt tolerance of wheat (Table 1 and Figure 4). Meanwhile, in wheat growing under normal water supply, *BADH-A1b* had no effect on the agronomic traits, which indicated excellent growth performance (Supplementary Table S3). However, *BADH-A1b* may confer improvements measurable in some agronomic traits in wheat subject to drought and salt conditions, though further research is required to determine whether this is the case.

### Distribution of *TaBADH-A1* locus alleles in Chinese wheat

In Chinese wheat, the mini-core collection (population 4) accounts for 70% of the genetic representation. The two alleles of *TaBADH-A1* locus were almost equally represented in the wheat mini-core collection (population 4; Figure 5A). The genetic resources of the two alleles were present in all wheat regions of China. This might be due to the fact that wheat zones have not been dry for many years. Wheat breeding in China has experienced three stages since the 1950s: disease resistance and stable yield, dwarfing and high yield, and high yield and excellent quality (He et al., 2011). Therefore, improving the adaptability of wheat has been neglected. This has resulted in an extremely uneven distribution of the two studied alleles in 121 cultivars (lines; population 1): *BADH-A1a* accounts for 90% while *BADH-A1b* accounts for only 10% (Figure 5B). Improving wheat stress resistance and paying attention to water saving have been proposed since the 1980s (He et al., 2018). This purpose might well explain the absence of *BADH-A1b* prior to the 1980s (Table 2). Since 2011, research efforts focusing on the lasting resistance of wheat have increased, considering such aspects as drought resistance, heat resistance, and adaptability to other factors (He et al., 2011). Therefore, the favorable allele *BADH-A1b* should be continuously selected for wheat growth under future unpredictable climate deterioration.

In summary, we found seven haplotypes of the *TaBADH-A1* gene in wheat, the seven haplotypes divided with 254 bp indel into two alleles. The expression level of *BADH-A1b* was significantly higher than that of *BADH-A1a* under drought and salt stress. *BADH-A1b* can increase the betaine content of wheat and further enhance its drought resistance and salt tolerance. *BADH-A1b* had no effect on the agronomic traits of wheat grown under normal conditions. Notably, the favorable allele *BADH-A1b* has been neglected in the selection, but it should be continuously selected in the face of future climate deterioration. Our study provides evidence indicating that *BADH-A1b* is an excellent candidate allele for breeding to improve the drought resistance and salt tolerance of wheat.

## Data availability statement

The original contributions presented in the study are included in the article/Supplementary Material, further inquiries can be directed to the corresponding authors.

## Author contributions

XZ and JX conceived and designed the experiments. MY and YY performed the experiments, analyzed the data, and wrote the manuscript. YY, MZ, SG, NL, FW, SZ, and HZ revised the manuscript. All authors contributed to the article and approved the submitted version.

## Funding

This study was funded by the National Natural Science Foundation of China (32171992, 31671693 and 31260324), Key Research and Development Project of Shaanxi Province (2019ZDLNY04-05), Central Guidance on Local Science and Technology Development Fund of Mongolia Autonomous Region (ZY20200089), Program for Young Talents of Science and Technology in Universities of Inner Mongolia Autonomous Region (NJYT-19-A25), and Research Program of Science and Technology at Universities of Inner Mongolia Autonomous Region (NJZZ22132).

## Conflict of interest

The authors declare that the research was conducted in the absence of any commercial or financial relationships that could be construed as a potential conflict of interest.

## Publisher’s note

All claims expressed in this article are solely those of the authors and do not necessarily represent those of their affiliated organizations, or those of the publisher, the editors and the reviewers. Any product that may be evaluated in this article, or claim that may be made by its manufacturer, is not guaranteed or endorsed by the publisher.

## Footnotes

^1^ https://plants.ensembl.org/index.html

^2^ https://www.rosaceae.org/

^3^ https://bioinformatics.psb.ugent.be/webtools/plantcare/html/
