# Supplementary material for: Identification of TaBADH-A1 allele for improving drought resistance and salt tolerance in wheat (Triticum aestivum L.)
Source: Front Plant Sci. 2022 Aug 1;13:942359. doi: 10.3389/fpls.2022.942359 (PMC9376607; doi:10.3389/fpls.2022.942359)
Supplement: Supplementary file 1 [file Image_1.pdf]

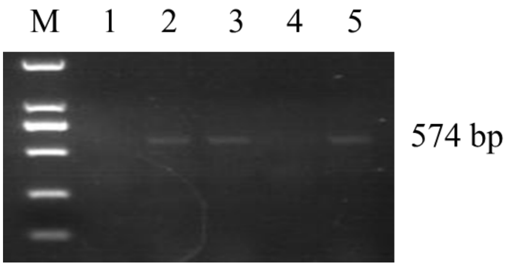

Figure S1. Location of molecular marker amplification on chromosome using in nulli-tetrasomic lines of Chinese Spring. M: DNA marker; 1: N6A-T6B; 2: N6B-T6D; 3: N6D-T6B; 4: H_2O; 5: Chinese Spring.
