# Supplementary material for: Identification of TaBADH-A1 allele for improving drought resistance and salt tolerance in wheat (Triticum aestivum L.)
Source: Front Plant Sci. 2022 Aug 1;13:942359. doi: 10.3389/fpls.2022.942359 (PMC9376607; doi:10.3389/fpls.2022.942359)
Supplement: Supplementary file 2 [file Image_2.pdf]

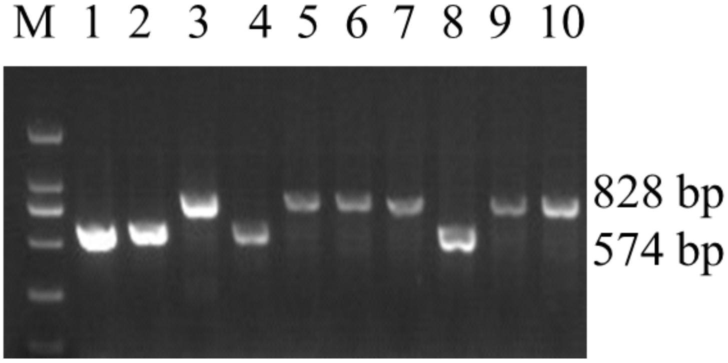

Figure S2. Amplified fragment length polymorphism amplification and analysis of the molecular marker. M: DNA marker; 1–10: Taishan 1, Chinese Spring, Bima 4, Longfumai 18, Yanfu 188, Ning 9940, Xinong 6028, Longmai 26, Jinmai 47, Zhongmai 9. All populations were tested, and only partial results are shown in the figure.
