# Supplementary material for: Identification of TaBADH-A1 allele for improving drought resistance and salt tolerance in wheat (Triticum aestivum L.)
Source: Front Plant Sci. 2022 Aug 1;13:942359. doi: 10.3389/fpls.2022.942359 (PMC9376607; doi:10.3389/fpls.2022.942359)
Supplement: Supplementary file 3 [file Image_3.pdf]

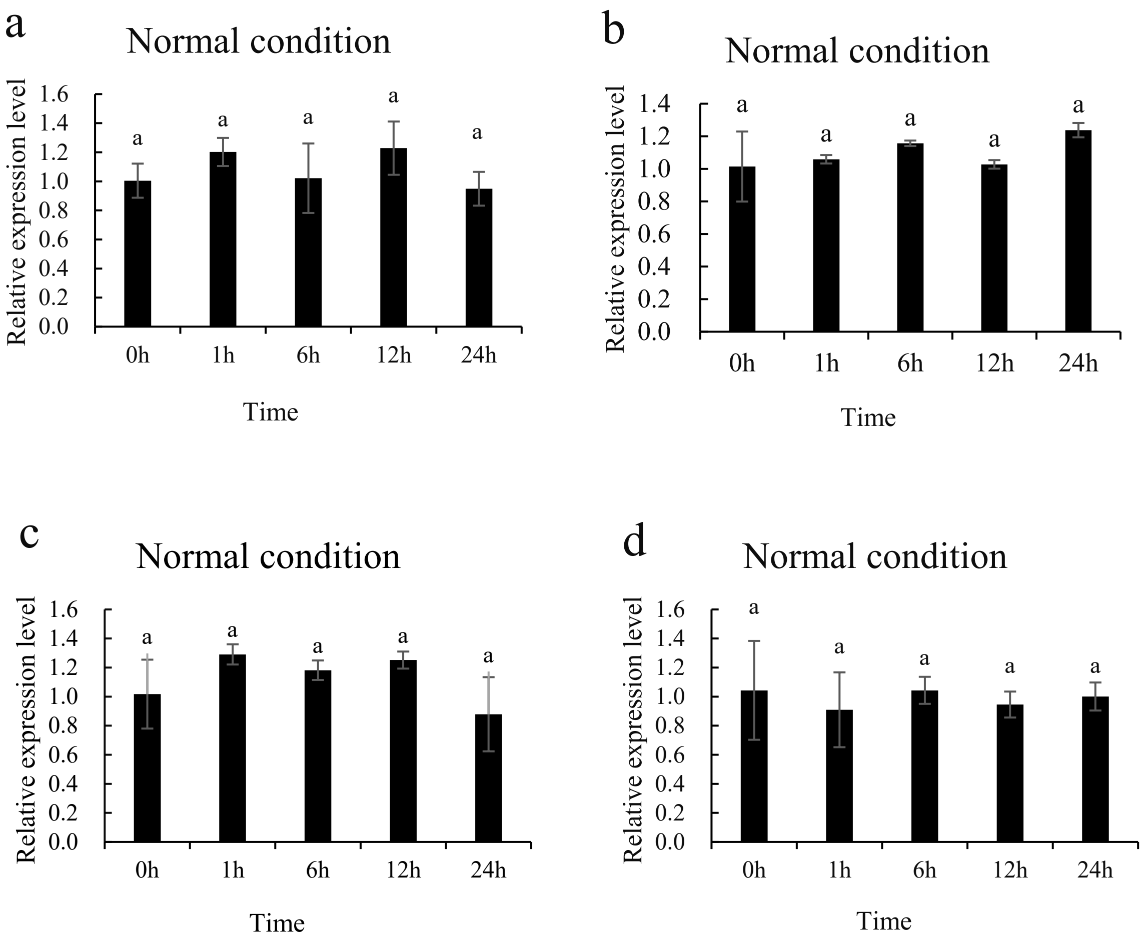

Figure S3. Expression patterns of *TaBADH-A1* locus alleles in normal condition. At 4-leaf stage, leaves were used as samples for RNA extraction. a: Taishan 1, b: Chinese Spring, c: Bima 4, d: Yanfu 188. The $2^{-\Delta\Delta CT}$ method was used to calculate relative gene expression. Statistically significant differences are indicated with different letters (LSD, $P < 0.05$).
