# Supplementary material for: Identification of TaBADH-A1 allele for improving drought resistance and salt tolerance in wheat (Triticum aestivum L.)
Source: Front Plant Sci. 2022 Aug 1;13:942359. doi: 10.3389/fpls.2022.942359 (PMC9376607; doi:10.3389/fpls.2022.942359)
Supplement: Supplementary file 4 [file Table_1.DOCX]

**Table S1.** Details of primers used in this study.

| Primer name | Primer sequence (5’-3’) | Tm (℃) | Function |
| --- | --- | --- | --- |
| C-F | GGTTGGTAGCGTTAAACACAGAATG | 62 | Cloned code and promoter region sequence of *TaBADH-A1* |
| C-R | GGGGGTTGCATTATACATTGGC |  |  |
| qPCR-F | ACTCCCCGTTAATATCCCAGTCT | 60 | qRT-PCR |
| qPCR -R | AGAGCCTGGTTTTGGAAGTCTTT |  |  |
| 6AM-F | CGCAAGTCAATGGGCTGTGTAT | 62 | Molecular marker 6AM |
| 6AM-R | CAAGACAGGAGGGGAATGGGAAAT |  |  |
