# Supplementary material for: Identification of TaBADH-A1 allele for improving drought resistance and salt tolerance in wheat (Triticum aestivum L.)
Source: Front Plant Sci. 2022 Aug 1;13:942359. doi: 10.3389/fpls.2022.942359 (PMC9376607; doi:10.3389/fpls.2022.942359)
Supplement: Supplementary file 6 [file Table_3.docx]

**Table S3.** Two alleles of *TaBADH-A* locus associations with agronomic traits in two recombinant inbred lines (populations 2 and 3).

Population 2

| Allele | GN  (number) | ESN  (number) | SL  (cm) | FLA  (cm^2^) | LLI  (cm) | PH  (cm) | TKW  (g) |
| --- | --- | --- | --- | --- | --- | --- | --- |
| *BADH-A1a* | 47.9±16.0 a | 18.9±3.3 a | 10.4±2.2 a | 30.5±12.8 a | 31.3±8.3 a | 114.5±10.4 a | 30.0±6.7 a |
| *BADH-A1b* | 53.5±17.4 a | 19.6±3.8 a | 11.1±1.8 a | 29.2±9.8 a | 31.3±5.4 a | 116.6±8.6 a | 30.2±6.9 a |

Population 3

| Alleles | GN  (number) | ESN  (number) | SL  (cm) | FLA  (cm^2^) | LLI  (cm) | PH  (cm) | TKW  (g) |
| --- | --- | --- | --- | --- | --- | --- | --- |
| *BADH-A1a* | 50.4±11.3 a | 18.3±1.7 a | 9.5±1.3 a | 26.0±7.6 a | 28.5±5.7 a | 108.5±10.0 a | 31.8±5.2 a |
| *BADH-A1b* | 47.4±9.4 a | 18.8±2.2 a | 9.6±1.4 a | 25.0±5.9 a | 28.1± 5.7 a | 112.8±9.8 a | 32.1±5.6 a |

Forty-eight lines were selected from each allele in populations 2 and 3. GN, grain number per spike; ESN, effective spikelet number per spik; TKW, thousand kernel weight; FLA, flag leaf area per plant; SL, spike length; LLI, length of the last internode; PH, plant height; Statistically significant differences are indicated with different letters (LSD, *P* < 0.05).
